# Association between alkaline phosphatase and hypertension in a rural Japanese population: The Nagasaki Islands study

**DOI:** 10.1186/1880-6805-32-10

**Published:** 2013-06-27

**Authors:** Yuji Shimizu, Mio Nakazato, Takaharu Sekita, Koichiro Kadota, Hironori Yamasaki, Noboru Takamura, Kiyoshi Aoyagi, Yosuke Kusano, Takahiro Maeda

**Affiliations:** 1 Department of Community Medicine, Nagasaki University Graduate School of Biomedical Science, Nagasaki, Japan; 2 Department of Island and Community Medicine, Nagasaki University Graduate School of Biomedical Science, Nagasaki, Japan; 3 Center for Health and Community Medicine, Nagasaki University, Nagasaki, Japan; 4 Department of Global Health, Medicine and Welfare, Nagasaki University Graduate School of Biomedical Sciences, Nagasaki, Japan; 5 Department of Public Health, Nagasaki University Graduate School of Biomedical Sciences, Nagasaki, Japan; 6 Department of Community Development, Nagasaki Wesleyan University, Nagasaki, Japan

**Keywords:** Alkaline Phosphatase, Drinking Status, Hypertension, Risk, Cross-sectional Study

## Abstract

**Background:**

Although serum alkaline phosphatase (ALP) levels have been associated with hypertension, and ALP is known as an enzyme affected by alcohol consumption, no study has been published on the associations between ALP and the risk of hypertension in relation to drinking status.

**Methods:**

We conducted a cross-sectional study of 2,681 participants (837 men and 1,846 women) aged 30 to 89 years undergoing a general health check-up to investigate the associations between ALP and hypertension in relation to drinking status.

**Results:**

Of the 2,681 participants, 1,549 (514 men and 1,035 women) were diagnosed with hypertension. A sex difference was observed for the relationship between ALP and hypertension. While no significant association was observed for men, the association was significantly positive for women. The multivariable adjusted odds ratio and 95% coincidence interval (CI) of hypertension per increment of 1-log ALP were 0.95 (95% CI: 0.56 to 1.59) for men and 1.57 (95% CI: 1.07 to 2.33) for women. When this analysis was restricted to nondrinkers, a significantly elevated risk of hypertension was observed for men and remained significant for women; that is, 3.32 (95% CI: 1.38 to 8.02) for men and 1.68 (95% CI: 1.11 to 2.55) for women.

**Conclusion:**

ALP is associated with hypertension for both male and female nondrinkers, but not for drinkers. For analyses of associations between ALP and blood pressure, alcohol consumption should thus be considered a potential confounder.

## Introduction

The alkaline phosphatase (ALP) enzyme catalyzes the hydrolysis of inorganic pyrophosphate [1], which is an inhibitor of vascular calcification [2]. While ALP is expressed in a variety of tissues, its concentrations are notably high in bone, liver, and kidneys [1]. One previous sex-combined study reported that ALP was significantly associated with hypertension [3], and another study found a weak but statistically significant association of ALP with blood pressure [4]. On the other hand, serum ALP levels are influenced by alcohol consumption [5,6], which has been positively associated with hypertension [7]. However, no study of the relationships between ALP and hypertension stratified by drinking status has been published. Since a previous study of ours established that alcohol consumption has a strong effect on the relationship between ALP and incidence of stroke in a general Japanese population [8], we hypothesized that the associations between ALP and hypertension might also be influenced by alcohol consumption. Since it is known that Japanese men are characterized by a high prevalence of current drinking and Japanese women by a low prevalence [8-10], a Japanese population would be a useful tool for studying this hypothesis. To examine this hypothesis, we analyzed general health check-up results for risk of hypertension for Japanese men and women.

## Methods

### Subjects

Written consent forms were available in Japanese to ensure comprehensive understanding of the study objectives, and informed consent was signed by the participants. This study was approved by the Ethics Committee for Use of Humans of Nagasaki University (project registration number 0501120073).

The source population included 3,883 residents of the western rural community of the Goto Islands (1,355 men and 2,528 women) 30 to 89 years old, who participated in this study between 2005 and 2010. Data for blood pressure were missing for 11 subjects (3 men and 8 women), data for drinking status missing for 69 subjects (23 men and 46 women), data for serum missing for 947 subjects (422 men and 525 women), and with history of cardiovascular disease for 175 subjects (72 men and 103 women) so a total of 1,202 subjects were excluded from this study. There were no differences in cardiovascular disease risk factors between participants for whom blood data were available and those for whom they were not. The remaining 2,681 participants, 835 men with a mean age of 64.5 years (± 10.5 standard deviation; range 30 to 89) and 1,846 women with a mean age of 62.5 years (± 11.3 standard deviation; range 30 to 89), were enrolled in this study.

### Data collection and laboratory measurements

Trained interviewers obtained information on smoking status, drinking status, medical history, as well as use of antihypertensive agents and medication for diabetes mellitus. Body weight and height were measured with an automatic body composition analyzer (BF-220; Tanita, Tokyo, Japan) when blood samples were obtained. Systolic and diastolic blood pressures were recorded at rest. Hypertension was defined as systolic blood pressure ≥140 mmHg and/or diastolic blood pressure ≥90 mmHg and/or use of antihypertensive medication.

Following collection of blood samples after overnight fasting, serum and plasma were separated and stored at −20°C and −80°C, respectively, until assay.

Serum concentrations of triglyceride (TG), hemoglobin A1c (HbA1c), aspartate transaminase (AST), γ-glutamyltranspeptidase (γ-GTP), ALP and serum creatinine were measured with standard laboratory procedures. The glomerular filtration rate (GFR) was estimated by means of an established method with three variations recently proposed by a working group of the Japanese Chronic Kidney Disease initiative [11]. 

According to this adapted version:

$$\begin{array}{l} \mathrm{GFR}\left(\mathrm{ml}/\text{minute}/1.73\;m^{2}\right)=194\\ \;\times {\left(\text{serum}\;\text{creatinine}\;\left(\text{enzyme}\;\text{method}\right)\right)}^{-1.094}\\ \;\times {\left(\mathrm{age}\right)}^{-0.287}\times \left(0.739\;\mathrm{for}\;\text{women}\right) \end{array}$$

HbA_1c_(National Glycohemoglobin Standardization Program) was calculated with the following equation recently proposed by a working group of the Japanese Diabetes Society [12]:

$$\begin{array}{l} {\mathrm{HbA}}_{1c}\left(\text{National}\;\text{Glycohemoglobin}\;\text{Standardization}\;\text{Program}\right)\\ \;={\mathrm{HbA}}_{1c}\left(\text{Japanese}\;\text{Diabetes}\;\text{Society}\right)+0.4\% \end{array}$$

Diabetes was defined as HbA_1c_(National Glycohemoglobin Standardization Program) ≥6.5%, and/or initiation of glucose-lowering medication or insulin therapy [13].

### Statistical analysis

Since previous studies including ours have demonstrated that Japanese men show a high prevalence of current drinkers and Japanese women a low prevalence [8,9,14], and since alcohol consumption has been positively associated with hypertension [7], we performed a sex-specific analysis.

Analysis of variance or logistic regression models were used to calculate differences in age-adjusted mean values or prevalence of potential confounding factors in relation to ALP levels. The subjects were divided into two groups according to smoking status (current smoker or not), alcohol consumption, (current drinker or not), diabetes (no, yes) or thyroid disease (no, yes). triglycerides (mg/dl), AST (IU/l), γ-GTP (IU/l) and GFR (ml/minute/1.73 m^2^) were adjusted as continuous variables. Covariance or general linear models were used to analyze differences in sex-specific, age-adjusted mean values for cardiovascular risk factors in relation to ALP levels, and logistic regression models to calculate odds ratios (OR) and 95% confidence intervals (CIs) of hypertension associated with ALP levels. Two different approaches were used for adjustments for confounding factors. The first adjustment was only for age, and the second consisted of other possible confounding factors; that is, smoking status (never smoker, former smoker, current smoker), alcohol consumption (nondrinker, current light to moderate drinker (1 to 6 times/week) and current heavy drinker (every day)), diabetes (no, yes), thyroid disease (no, yes), triglycerides (mg/dl), AST (IU/l), γ-GTP (IU/l) and GFR. Because ALP levels are strongly affected by drinking status [5,6], we also investigated associations between ALP and hypertension among nondrinkers.

All statistical analyses were performed with the SAS system for Windows (version 9.3; SAS Inc., Cary, NC, USA). All *P* values for statistical tests were two-tailed, and *P* <0.05 was regarded as statistically significant.

## Results

Of the 2,681 participants (835 men and 1,846 women), 1,549 (514 men and 1,035 men) were diagnosed with hypertension.

Table 1 presents sex-specific age-adjusted baseline characteristics by ALP level. For both men and women, triglycerides and γ-GTP showed significantly positive associations with ALP levels. For men, the prevalence of current drinkers was inversely associated with ALP levels, and for women systolic blood pressure, diastolic blood pressure, antihypertensive medication use and AST were positively and significantly associated with ALP levels.

**Table 1:** **Age**-**adjusted mean values and proportions by alkaline phosphatase levels**

	**ALP quartiles**				
	**Q1 (low)**	**Q2**	**Q3**	**Q4 (high)**	***P *value**
Men					
Median values for serum ALP (U/l)	172	209	250	318	
Number at risk	205	215	207	208	
Age (years)	63.9 ± 10.0	63.2 ± 11.5	65.3 ± 10.2	65.7 ± 10.2	
Systolic blood pressure (mmHg)	140	142	143	141	0.504
Diastolic blood pressure (mmHg)	84	85	86	85	0.390
Antihypertensive medication use (%)	27	22	25	26	0.735
Thyroid disease (%)	0.0	0.5	1.0	0.0	0.300
Body mass index (kg/m^2^)	23.7	23.8	23.6	23.3	0.337
Current drinker (%)	62	53	46	45	0.002
Current smoker (%)	20	23	27	29	0.105
Serum triglycerides (mg/dl)	128	138	153	142	0.044
Diabetes (%)	8	7	11	12	0.267
Serum aspartate aminotransferase (AST) (IU/l)	24	25	25	26	0.061
Serum γ-glutamyltranspeptidase (γ-GTP ) (IU/l)	36	41	51	60	<0.001
Serum creatinine, (mg/dl)	0.90	0.93	0.92	0.91	0.562
Glomerular filtration rate (ml/minute/1.73 m^2^)	70.3	68.4	68.5	70.7	0.411
Women					
Median values for serum ALP (U/l)	170	220	263	337	
Number at risk	465	458	467	456	
Age (years)	56.4 ± 12.8	63.7 ± 10.8	64.3 ± 9.6	65.8 ± 9.2	
Systolic blood pressure (mmHg)	137	140	141	142	<0.001
Diastolic blood pressure (mmHg)	80	83	84	84	<0.001
Antihypertensive medication use (%)	26	25	24	31	0.029
Thyroid disease (%)	0.4	0.7	1.3	0.9	0.534
Body mass index (kg/m^2^)	22.6	22.9	23.2	23.1	0.083
Current drinker (%)	13.3	13.4	10.2	11.1	0.338
Current smoker (%)	3.6	3.3	4.7	4.5	0.682
Serum triglycerides (mg/dl)	113	118	127	133	<0.001
Diabetes (%)	5.8	5.4	5.6	7.1	0.689
Serum aspartate aminotransferase (AST) (IU/l)	21.4	21.9	22.0	23.1	0.011
Serum γ-glutamyltranspeptidase (γ-GTP)IU/l)	20	24	25	31	<0.001
Serum creatinine, (mg/dl)	0.724	0.715	0.716	0.724	0.740
Glomerular filtration rate (ml/minute/1.73 m^2^)	66.7	67.7	67.7	66.5	0.574

Age presented as mean ± standard deviation. Serum alkaline phosphatase (ALP) levels for quartiles were <194 U/l, 194 to 228 U/l, 229 to 275 U/l, and >275 U/l for men and <199 U/l, 199 to 240 U/l, 241 to 290 U/l, and >290 U/l for women.

Table 2 presents the OR and 95% CIs for hypertension according to ALP levels. While no significant association was observed for men, it was significantly positive for women. The multivariable adjusted OR and 95% CI for hypertension per increment of 1-log ALP were 0.95 (95% CI: 0.56 to 1.59) for men and 1.57 (95% CI: 1.07 to 2.33) for women.

**Table 2:** **Sex**-**specific odds ratio and 95**% **confidence interval for hypertension in relation to alkaline phosphatase levels**

	**ALP quartiles**					**1 SD increments in ALP**	**Log ALP**
	**Q1 (low)**	**Q2**	**Q3**	**Q4 (high)**	***P *for trend**		
Men							
Number at risk	205	215	207	208			
Number of cases (%)	118 (58)	134 (62)	134 (65)	128 (62)			
Age-adjusted OR	1.00	1.26 (0.85 to 1.88)	1.30 (0.87 to 1.95)	1.11 (0.75 to 1.66)	0.590	1.00 (0.87 to 1.16)	1.00 (0.62 to 1.59)
Multivariable OR	1.00	1.30 (0.86 to 1.99)	1.29 (0.84 to 1.99)	1.16 (0.75 to 1.79)	0.535	0.98 (0.83 to 1.15)	0.95 (0.56 to 1.59)
Women							
Number at risk	465	458	467	456			
Number of cases (%)	186 (40)	271 (59)	273 (58)	305 (67)			
Age-adjusted OR	1.00	1.33 (0.98 to 1.78)	1.22 (0.91 to 1.64)	1.61 (1.19 to 2.17)	0.006	1.21 (1.09 to 1.35)	1.81 (1.26 to 2.60)
Multivariable OR	1.00	1.29 (0.95 to 1.76)	1.14 (0.84 to 1.55)	1.49 (1.09 to 2.04)	0.041	1.17 (1.04 to 1.31)	1.57 (1.07 to 2.33)

Multivariable odds ratio (OR) adjusted further for age, body mass index, smoking, alcohol intake, diabetes, thyroid disease, serum triglycerides, serum aspartate aminotransferase (AST) , serum γ-glutamyltranspeptidase (γ-GTP) and glomerular filtration rate. Hypertension defined as systolic blood pressure ≥140mmHg and/or diastolic blood pressure ≥90 mmHg and/or use of antihypertensive agents. Serum alkaline phosphatase (ALP) levels for quartiles were <194 U/l, 194 to 228 U/l, 229 to 275 U/l, and >275 U/l for men and <199 U/l, 199 to 240 U/l, 241 to 290 U/l, and >290 U/l for women.

We also investigated the sex-specific influence of drinking status on ALP levels and found a significantly inverse association between ALP and drinking status (nondrinker, current light-to-moderate drinker (1 to 6 times/week), and current heavy-drinker (every day)) for men, while essentially the same tendency was observed for women but without reaching statistical significance. For men, the age-adjusted mean values of ALP were 254 for nondrinkers, 235 for current light-to-moderate drinkers, and 233 for current heavy drinkers (*P* = 0.003), and for women the corresponding values were 252, 247, and 240 (*P* = 0.292).

We also evaluated the risk of hypertension for drinkers. Drinking status for men showed a significant association with risk of hypertension but not so for women. The age-adjusted OR and 95% CI of hypertension for drinking status (nondrinker or current drinker) were 1.70 (95% CI: 1.28 to 2.27; *P* <0.001) for men and 1.15 (95% CI: 0.84 to 1.57; *P* = 0.385) for women.

Table 3 presents the ORs and 95% CIs for hypertension by ALP level in relation to drinking status. Significantly positive associations were observed for nondrinkers, but not for drinkers. For nondrinkers the adjusted OR and 95% CI for hypertension per increment of 1-log ALP were 3.32 (95% CI: 1.38 to 8.02) for men and 1.68 (95% CI: 1.11 to 2.55) for women, and for drinkers the corresponding values were 0.42 (95% CI: 0.20 to 0.89) for men and 1.03 (95% CI: 0.30 to 3.50) for women.

**Table 3:** Sex-specific data for hypertension in relation to alkaline phosphatase levels and drinking status

	**ALP quartiles**					**1 SD increments in ALP**	**Log ALP**
	**Q1 (low)**	**Q2**	**Q3**	**Q4 (high)**	***P *for trend**		
Nondrinker men							
Number at risk	77	100	112	115			
Number of cases (%)	35 (45)	50 (50)	69 (62)	73 (63)			
Age-adjusted OR	1.00	1.27 (0.69 to 2.34)	1.91 (1.04 to 3.48)	1.98 (1.09 to 3.62)	0.011	1.40 (1.10 to 1.79)	2.96 (1.33 to 6.58)
Multivariable OR	1.00	1.37 (0.71 to 2.64)	1.96 (1.03 to 3.73)	2.23 (1.16 to 4.28)	0.009	1.46 (1.12 to 1.91)	3.32 (1.38 to 8.02)
Nondrinker women							
Number at risk	390	399	423	412			
Number of cases (%)	162 (42)	238 (60)	250 (59)	277 (67)			
Age-adjusted OR	1.00	1.34 (0.97 to 1.84)	1.27 (0.93 to 1.73)	1.64 (1.19 to 2.27)	0.006	1.22 (1.08 to 1.37)	1.84 (1.24 to 2.72)
Multivariable OR	1.00	1.33 (0.96 to 1.85)	1.21 (0.87 to 1.67)	1.58 (1.12 to 2.21)	0.023	1.19 (1.05 to 1.35)	1.68 (1.11 to 2.55)
Drinker men							
Number at risk	128	115	95	93			
Number of cases (%)	83 (65)	84 (73)	65 (68)	55 (59)			
Age-adjusted OR	1.00	1.53 (0.88 to 2.67)	1.14 (0.64 to 2.02)	0.76 (0.44 to 1.33)	0.295	0.82 (0.67 to 1.00)	0.59 (0.31 to 1.14)
Multivariable OR	1.00	1.39 (0.78 to 2.50)	0.94 (0.51 to 1.73)	0.62 (0.34 to 1.14)	0.103	0.73 (0.58 to 0.91)	0.42 (0.20 to 0.89)
Drinker women							
Number at risk	75	59	44	44			
Number of cases (%)	24 (32)	33 (56)	23 (52)	28 (64)			
Age-adjusted OR	1.00	1.17 (0.52 to 2.66)	0.87 (0.36 to 2.12)	1.34 (0.54 to 3.28)	0.711	1.16 (0.87 to 1.55)	1.60 (0.58 to 4.41)
Multivariable OR	1.00	0.89 (0.36 to 2.20)	0.70 (0.26 to 1.87)	1.14 (0.42 to 3.09)	0.953	1.01 (0.68 to 1.49)	1.03 (0.30 to 3.50)

Data presented as odds ratio (95% confidence interval). Multivariable odds ratio (OR) adjusted further for age, body mass index, smoking, alcohol intake, diabetes, thyroid disease, serum triglycerides, serum aspartate aminotransferase (AST), serum γ-glutamyltranspeptidase (γ-GTP) and glomerular filtration rate. Hypertension defined as systolic blood pressure ≥140 mmHg and/or diastolic blood pressure ≥90 mmHg and/or use of antihypertensive agents. Serum alkaline phosphatase (ALP) levels for quartiles were <194 U/l, 194 to 228 U/l, 229 to 275 U/l, and >275 U/l for men and <199 U/l, 199 to 240 U/l, 241 to 290 U/l, and >290 U/l for women.

Another analysis was performed to evaluate associations between serum ALP levels and systolic and diastolic blood pressure. We found that ALP levels were independently positively associated with both systolic and diastolic blood pressure for nondrinkers but not for drinkers. The results of multiple (including antihypertensive medication use) linear regression analysis of systolic and diastolic blood pressure per increment of 1-log ALP for nondrinkers were regression coefficient β = 2.10 (95% CI: 1.08 to 4.09; *P* = 0.028) and β = 3.54 (95% CI: 1.81 to 6.92; *P* <0.001) for men, and β = 1.62 (95% CI: 1.19 to 2.21; *P* = 0.002) and β = 2.02 (95% CI: 1.48 to 2.75; *P* <0.001) for women, while for drinkers the corresponding values were β = 0.96 (95% CI: 0.57 to 1.64; *P* = 0.884) and β = 0.62 (95% CI: 0.37 to 1.06; *P* = 0.082) for men, and β = 0.80 (95% CI: 0.34 to 1.91; *P* = 0.619) and β = 0.91 (95% CI: 0.38 to 2.16; *P* = 0.822) for women, respectively.

We also identified a significant effect on hypertension of interaction between ALP levels and drinking status (nondrinkers or current drinkers) for men but not for women. The multivariable-adjusted *P* value of this effect of interaction on hypertension was 0.002 for men and 0.900 for women.

## Discussion

Our findings demonstrate that serum ALP is associated with hypertension for both male and female nondrinkers, but not for drinkers. For analyses of associations between ALP and blood pressure, alcohol consumption should thus be considered a potential confounder.

A previous study of 4,155 men and women conducted by the United States National Health and Nutrition Examination Survey reported that ALP showed a significant association with higher frequency of hypertension (*P* = 0.01). Moreover, compared with the lowest quartiles of ALP, the adjusted OR associated with the highest quartiles was 1.6 (95% CI: 1.0 to 2.5) [3]. Another study of 79 South African men reported that 24-hour systolic blood pressure was positively associated (regression coefficient β= 0.289, *P* = 0.018) with serum ALP [15].

Although no such significant association was observed for men in our study, a significantly positive association was observed for women. We found further that, when these analyses were restricted to nondrinkers, the association became significantly positive for men and remained significant for women. However, no such significant positive associations were observed for either male or female drinkers.

Previous studies have reported that ALP levels are affected by alcohol consumption [5,6]. A Danish general population study reported that the amount of alcohol intake was inversely and significantly associated with ALP levels for both men and women [5]. Our study also showed that ALP levels were significantly associated with drinking status for men, and that for women, even though the association was not statistically significant, essentially the same tendency was observed. Further, alcohol consumption is positively associated with risk of hypertension [7]. NIPPON DATA 90 reported that a large percentage of hypertensive subjects in a general Japanese male population suffered from alcohol-induced hypertension [14]. This is compatible with our study’s findings that drinking status is a significant risk factor for hypertension for men but not for women. The association between ALP levels and hypertension may this be seriously confounded by alcohol consumption, especially for Japanese men. The significant effect on hypertension of the interaction between ALP levels and drinking status (nondrinker or current drinker) identified for men but not for women may be induced by such drinking-related mechanisms.

The mechanisms for the positive association of higher ALP levels with risk of hypertension have not yet been elucidated. Tonelli and colleagues reported identifying an independent relationship between higher levels of ALP and overall mortality for survivors of myocardial infarction and in a general population sample. In their study, systolic blood pressure was positively associated with ALP levels [16]. Orita and colleagues found a positive correlation between serum total ALP activity and bone-type ALP activity in mouse calcified vascular lesions [17]. Another study demonstrated the presence of bone-type ALP in human vascular smooth muscle cells [18], which suggests that a higher level of bone-type ALP activity may accelerate the development of cardiovascular events through cardiovascular calcification, which is also positively associated with hypertension. Jensky and colleagues reported that among 9,510 participants (42.5% women) who underwent electron beam computed tomography scanning as part of a routine health maintenance scanning, systolic hypertension features a robust and important significant correlation with calcified atherosclerosis [19].

Another possible mechanism for the association between ALP levels and risk of hypertension is impaired vascular homeostasis, since hematopoietic stem cells derived from bone marrow play a major role in vascular homeostasis [20-22]. Since osteoblasts, whose activity can be assessed by bone-type ALP expression [1,23], regulate the production of hematopoietic stem cells in bone marrow [24-26], serum ALP levels may correlate with vascular homeostatic activity. Some previous studies have reported that hematopoietic stem cells participate in the pathogenesis of atherosclerosis [27] and promote angiogenesis [20], while other studies established that vascular maturation and stabilization were end stages of neovascularization and angiogenesis [28]. Higher ALP levels may thus indicate progression of atherosclerosis. Yet another study showed that elevated ALP levels were associated with atherosclerosis, which leads to peripheral arterial disease and was assessed using the ankle-brachial blood pressure index, and that this association was independent of other traditional cardiovascular risk factors [29]. Furthermore, hypertension is not only a well-established cardiovascular risk factor but also increases the risk of atherosclerosis [30]. Elevated ALP levels may therefore constitute a risk for hypertension due to progressive atherosclerosis. A previous study of ours, the Circulatory Risk in Community Study, detected a significantly positive association between ALP levels and blood pressure (either systolic and/or diastolic) for both men and women, which may induce these mechanisms [8]. On the other hand, the nature of those mechanisms also indicated that elevated ALP levels might perform an important role as direct surrogate markers for progressive atherosclerosis and blood pressure as an indirect surrogate marker for atherosclerosis. In this respect, previous studies, even after adjustments for systolic blood pressure and antihypertensive medication use, showed significant associations between ALP level and mortality [16], and incidence of stroke [8].

The same mechanisms also point to the clinical relevance of this study’s finding that among ALP can be used for nondrinkers to evaluate risk of hypertension or progression of atherosclerosis, which might be useful for controlling blood pressure.

This study has certain potential limitations, which warrant consideration. First, because no measurements of the ALP isozyme were performed [31], we could not assess which type of ALP was associated with the risk of hypertension. Second, we did not have access to menopausal status data, which may have affected ALP levels [32], but a significantly positive association was observed between ALP levels and risk of hypertension for nondrinking women. Further investigations using menopausal data are therefore necessary. Third, only limited data for thyroid disease were available, which may have confounded the associations between ALP and risk of hypertension [33]. Nevertheless, we found a significant association for both nondrinking men and women, even though the prevalence of thyroid disorder in the general Japanese population reportedly shows a strong sex difference [34]. Fourth, even though a previous Japanese study reported that, compared with never-drinkers, former male drinkers men showed a significantly higher risk of cardiovascular disease but their female counterparts did not [10], we could not conduct an analysis of never-drinkers because data for this group were not available. However, we found a significantly positive association between ALP levels and hypertension for both nondrinking men and women. Although the association between ALP levels and risk of hypertension was shown to be independent of the traditional risk factors, we did not adjusted for other potential confounders whose values were associated with ALP, such as calorie, protein, vitamin C, magnesium, and zinc deficiencies [35], and drinking volume of alcohol consumption.

In conclusion, serum ALP is associated with hypertension for both male and female nondrinkers, but not for drinkers. For analyses of associations between ALP and blood pressure, alcohol consumption should thus be considered a potential confounder.

## Abbreviations

ALP: Alkaline phosphatase; AST: Aspartate transaminase; CI: Confidence interval; GFR: Glomerular filtration rate; γ-GTP: γ-Glutamyltranspeptidase; HbA1c: Hemoglobin A1c; OR: Odds ratio.

## Competing interests

The authors declare that they have no competing interests.

## Authors’ contributions

All authors read and approved the final manuscript. YS carried out the design of the study and performed the statistical analysis, interpreted the data, and drafted or revised the manuscript. MN, TS and KK designed the study, were involved in data collection, and checked the manuscript. HY, NT, KA, and YK participated in the study concept and checked the manuscript. TM was a general coordinator and designed the study.
